# How do Registered Nurses Understand Followership?

**DOI:** 10.1177/08445621231173793

**Published:** 2023-05-09

**Authors:** Deena M. Honan, Noelle Rohatinsky, Gerri Lasiuk

**Affiliations:** 1 38682 Northwestern Polytechnic, Grande Prairie, Alberta, Canada; 2 College of Nursing, 7235 University of Saskatchewan, Saskatoon, Saskatchewan, Canada; 3 College of Nursing, 7235 University of Saskatchewan, Regina, Saskatchewan, Canada

**Keywords:** Followership, registered nurse, team, grounded theory

## Abstract

**Background:**

Despite a consensus that followers and leaders are interdependent, the focus of nursing education, practice, and research has been leader centred. This has spawned calls in the nursing literature for increased scholarship on followership in nursing.

**Purpose:**

To develop a grounded theory of followership in nursing.

**Method:**

This study addressed the question - how do registered nurses understand followership? 11 registered nurses participated in online interviews that were later transcribed and analyzed following Charmaz's approach to Constructivist Grounded Theory.

**Results:**

The core category of trusting informal and formal leaders was co-constructed from the data. A conceptual model, titled Followership as Trust in Acute Care Nursing Teams, illustrates that the nurses’ decision to trust (and subsequently to engage in following) hinges on sharing the load (understanding one's role, accepting one's role, and working together); demonstrating knowledge (having experience, modelling, and mentoring); and connecting through communication (knowing the goal and communicating clearly). When participants fully trust formal and informal leaders, they engage in following as proactive members of the team, provide solutions to problems, and take initiative. Conversely, when they are less trusting of informal and formal leaders, they are less willing to follow.

**Conclusions:**

This study underscores the importance of trust between followers and leaders for effective team function and safe patient care. More research on the follower-leader dynamic in nursing is needed to inform education, policy, and practice so that every nurse possesses the knowledge and skill to be both a follower and a leader.

## Background and purpose

Teamwork is an essential aspect of nursing that affects patient care and quality outcomes (Crawford & Daniels, 2014; Kaiser & Westers, 2018; Kean et al., 2011; Polis et al., 2017; Whitehair et al., 2018; Whitlock, 2013). Teams consist of a leader and follower/s who must work together in a synergistic relationship to support each other, share responsibilities, and rely on each other's experiences and knowledge (Dickerson & Latina, 2017). Teams can be comprised of dyads or larger groups within the same profession or interprofessional groups. Leaders and followers cannot exist without each other; they are two sides of the same coin, interdependent in their relationship and powerful in their synergy (Chaleff, 2009; McCallum, 2013; Whitlock, 2013). Leading is an interactive process involving “three dynamic elements: a leader, a follower, and a situation” (Hoffart, 2017, p. 396). Within this context, followership is the willingness to cooperate in a coordinated way to accomplish shared goals while engaging in teamwork (Bastardoz & Van Vugt, 2019).

In their 2018 study, Kaiser and Westers noted that members of high functioning teams clearly understand their roles and responsibilities, work respectfully with each other, and do not rely as heavily on a leader for specific purpose-related instruction. This suggests that high functioning teams involve effective followership and while the leadership role has been extensively researched in nursing, there is a dearth of research about followership in nursing. Despite this gap, there is a recognition that followership is an important topic for the profession and discipline of nursing. For example, Miller (2007) wrote that without followers, there will not be much success in any activity, and she called for greater recognition of the importance of the role of followership, noting that nurses must be equipped with the knowledge and understanding of followership to fulfill that role.

The idea that followership is integral to teamwork was further advanced in an editorial by Guidera and Gilmore (1988) who claimed that followers are erroneously characterized as “apathetic, indecisive, or passive” (p. 1017). To the contrary, Guidera and Gilmore (1988) argued that followership warrants “equal time, respect, and study” (p. 1017) and that leaders and followers must work in alliance for each to be effective. They also observed that the characteristics of effective followers and leaders are quite similar and include “humility, assertiveness, courage, aspiration, trust, practicality, and determination” (p. 1017). The same authors also advocated that nursing education should focus on followership skills because following effectively is imperative in teamwork.

Another supporter of followership education in nursing, DiRienzo (1994), contends that followership involves knowledge, beliefs, attitudes, behaviours, and skills that can be learned and practiced and that followers and leaders “are separate yet equal” (p. 30). She believes “without effective followers in nursing, our leaders face severe limitations” (p. 26). Furthermore, she argues that followership should be included in baccalaureate curricula to support novice nurses with opportunities to mature in their knowledge and practice before taking on formal leadership positions. DiRienzo challenged nurse leaders and educators to correct the widespread misunderstanding of followership and to educate undergraduate and graduate students to be exemplary followers.

Every nurse has been in a follower role at some point in time; however, the focus of nursing research, education, and practice has been primarily on leadership, despite calls for increased attention to and scholarship on followership in nursing. For these reasons, the focus of this study is understanding nurses’ perceptions of followership.

### Purpose statement

The purpose of this study is to develop a theory of followership in nursing using constructivist grounded theory (Charmaz, 2014). The objectives are to understand followership in nursing within an acute care clinical environment by a) exploring registered nurses’ (RNs’) perceptions of followership; b) discover how nurses construct or co-construct followership; c) understand nurses’ schema regarding followership; and d) explore organizational features that influence RNs’ construction of followership.

The constructivist model of followership described by Carsten et al. (2010) guided the study. Motivated by the lack of scholarship on followership, Carsten et al. (2010) undertook a qualitative study to “deconstruct the meaning of followership” (p. 543). This work is pivotal because it begins to conceptualize how followership is experienced and enacted. Carsten et al. (2010) concluded that followership is socially constructed, and that the construction of followership depends on an individual's schema (beliefs, perceptions, behaviours) and the context (organizational climate and leadership style), which affect how individuals enact the follower role. Individuals construct their own unique understanding of followership, which is influenced by their schema and the context in which they work. There are as many social constructions of followership as there are individuals in follower roles (Meindl, 1995) and each one helps to inform a holistic understanding of the phenomenon. Constructivist grounded theory methodology is consistent with the constructivist model of followership as both view the construction of knowledge as a social process.

## Methods and procedures

### Study design

Charmaz's (2014) constructivist grounded theory (CGT) was employed to address the research question – how do registered nurses, who are members of a healthcare team in an acute care setting, understand followership? CGT is a qualitative approach that aims to construct an understanding of a social process of interest through inductive and abductive interpretation within the context of a naturalistic setting (Charmaz, 2014; Denzin & Lincoln, 2018).

### Setting and sample

The setting for this study was a tertiary care hospital in Western Canada. Participants were recruited from the hospital's four acute care units - two medical and two surgical units. Individuals were eligible to participate if they were registered with the College and Association of Registered Nurses of Alberta (CARNA), were employed as staff nurses in one of the target hospital's four designated units and provided informed consent. RNs employed in a supervisory position at the time of the interviews were excluded from participating.

Purposive sampling was initially used to recruit nine participants who agreed to reflect on the topic of followership and participate in a conversational interview (Polit & Beck, 2017). In CGT, the adequacy of the sample size is a judgement of the researcher and is based on the depth and richness of data (Creswell & Plano Clark, 2018) and approximation to theoretical saturation. Following a preliminary analysis, the determination was made to recruit additional participants and snowball sampling was used to recruit two more participants. The data was collected at the height of the COVID-19 pandemic, which undoubtably influenced the number of participants willing to volunteer for the study.

### Protection of human subjects

Ethics approval was obtained from the University of Saskatchewan Research Services and Ethics Office, the University of Alberta Health Research Ethics Board (which provides ethical oversight for Alberta Health Services), and Northwestern Polytechnic (formerly Grande Prairie Regional College) Research Ethics Board. Operational approval was obtained from the target hospital.

Participants provided informed written consent, which was affirmed verbally immediately prior to the start of the interview. Anonymity was not possible with interviews; however, to ensure confidentiality participants were assigned a numerical identifier and all identifying information was removed from the interview transcripts. Interview audio tapes, interview transcripts, memos, and signed consent forms, are stored in a locked cabinet in the first author's private office and all electronic data is being stored on a password protected computer and on a secure server (i.e., OneDrive).

### Data collection procedures

Initial email contact was made with the managers and clinical educators on the target units to explain the purpose of the study and request permission to recruit RNs. Once permission was received, recruitment posters and a participant information letter were placed in each unit's staff room inviting potential participants to contact the first author and to attend an online information session. Two such presentations were offered on different dates and times. In addition, a link to a video explaining the study was included on the poster for individuals who were unable to attend an online information session. Online rather than in-person information sessions were used for recruitment due to COVID-19 physical distancing requirements.

Digitally recorded, individual interviews were completed by the first author between August and October 2021 using a semi-structured interview guide. This short timeline for data collection was influenced by the pandemic and constraints on participants’ time. The interviews were conducted via telephone and lasted between 20 and 40 min each. Interviews were scheduled at a time selected by the participant and began with introductions, a review of the purpose of the study, an invitation to ask outstanding questions, and confirmation of the participant's consent. All participants had the opportunity to debrief following the interview.

Second interviews with the first two participants were completed before the remaining nine participants were interviewed. The information gleaned from these second interviews facilitated the creation of additional questions related to the concept of being open to followership and helped to strengthen theoretical sensitivity. Consistent with the practice of theoretical sampling, three participants took part in another interview to confirm whether they agreed with the findings of the study. The study's rigor was strengthened when these three participants endorsed the core category and agreed that the analysis resonated with their perceptions of followership.

Charmaz (2006) described four requirements for rigorous CGT studies – credibility, originality, resonance, and usefulness. Credibility for this study was achieved through close attention to the information provided by the participants and fidelity with the CGT method (Charmaz, 2006, 2014). The proposed grounded theory of followership is original, and answers calls in the literature for a greater understanding of followership in nursing and provides a new perspective on the integral, interdependent, and synergistic relationship between followers and leaders. Resonance reflects participants being able to make sense of the grounded theory presented to them and evokes a sense of agreement (Charmaz, 2006 ). Findings will help to inform an understanding of followership and the leader-follower dynamic.

### Data analysis procedures

Data collection and analysis proceeded concurrently to enable researchers to remain close to the participants’ understanding of their experiences and reflects on how followership was constructed and enacted (Charmaz, 2014). In keeping with the tenets of grounded theory research, memo writing was used to create a paper trail of key decisions related to data collection, analysis, coding, and theory creation (Cooney, 2011). Interviews were transcribed verbatim and cleaned in preparation for coding. Analysis was iterative and involved constant comparison of data, development of focused codes, theoretical codes, and reflexivity (Charmaz, 2014). Data was analysed within and between interview transcripts to identify themes and patterns across the data. The first step of data analysis was initial coding, utilizing gerunds (the verb form of nouns) to explain actions in a line-by-line process (Charmaz, 2014). The next step, focused coding, is a process of creating focused codes and developing a bigger picture of the data (Charmaz, 2014). As the analysis progressed, it became apparent that the focused codes were better suited as categories and the core category evolved into the theoretical code. Theoretical coding elucidated the relationships among the categories and decreased ambiguity regarding the construction of a preliminary conceptual model of followership (Charmaz, 2014). See Table 1 for coding examples.

**Table 1.** Coding examples.

Sub-categories	Exemplars	Categories	Core Category
Understanding one's role	“I feel like a follower should also understand their role prior to getting into it. They understand that they are under the umbrella of somebody else” (P6)	Sharing the load	Trusting informal and formal leaders 1. Trusting informal and formal leaders are sharing the load 2. Trusting informal and formal leaders are demonstrating knowledge 3. Trusting informal and formal leaders are connecting through communication 4. Trusting informal and formal leaders facilitates willingness to engage
Accepting one's role	“…being able to accept that role and not necessarily always feeling like you need to be in a leadership role” (P3)		
Working together	“…everyone works together because it's multiple members of the team to one patient” (P10)		
Having experience	“She just has kind of naturally taken over that role. She's been here the longest, she has the most experience in this department” (P2)	Demonstrating knowledge	
Modelling	“…they just lead by example, like they help out too…that makes me want to follow” (P4)		
Mentoring	“I would…just look up to them to hopefully follow in their footsteps one day” (P1)		
Knowing the goal	“…make sure that everyone is aware of what that goal is and that everyone is working towards that goal in the most effective way” (P2)	Connecting through communication	
Communicating clearly	“I think the biggest element of an effective team is communication” (P6)		
Learning	“I hope they would be open to new learning and to…learn from the person that they’re following” (P5)	Willingness to engage	
Being open	“…the attitude of a follower has to be… adaptable, flexible” (P10)		
Participating	“I see the followers as the people carrying out the actions, the doers” (P8)		

## Results

Eleven RNs participated in the study. All identified as female, their ages ranged from 20 to 49 years, and were employed in a casual, part-time, or full-time position. Participants included a newly graduated RN with two months of experience, four RNs with more than 15 years of nursing experience, and six others with between two and 14 years of experience. Ten participants were baccalaureate prepared and one had a master's degree in nursing. One participant acknowledged having worked in a manager position in the past. This wide range in ages and nursing experience provided multiple views of followership, which were influenced by participants’ formal education, personal beliefs, behaviours, expectations of other team members, and the organizational context. In this study, participant's understanding of followership is constructed by the team dynamic they work in and their understanding of the relationship between followers and leaders. Team members are considered the leader and the followers. A formal leader refers to someone officially appointed to a leadership role and an informal leader is a member of the team, a follower, who provides leadership within the team.

The four focused codes, sharing the load, demonstrating knowledge, connecting through communication, and willingness to engage, became the categories. Finally, the core category, trusting informal and formal leaders, was developed to illustrate the relationship among and between the categories created during focused coding and became the theoretical code (Charmaz, 2014).

**Figure 1.**
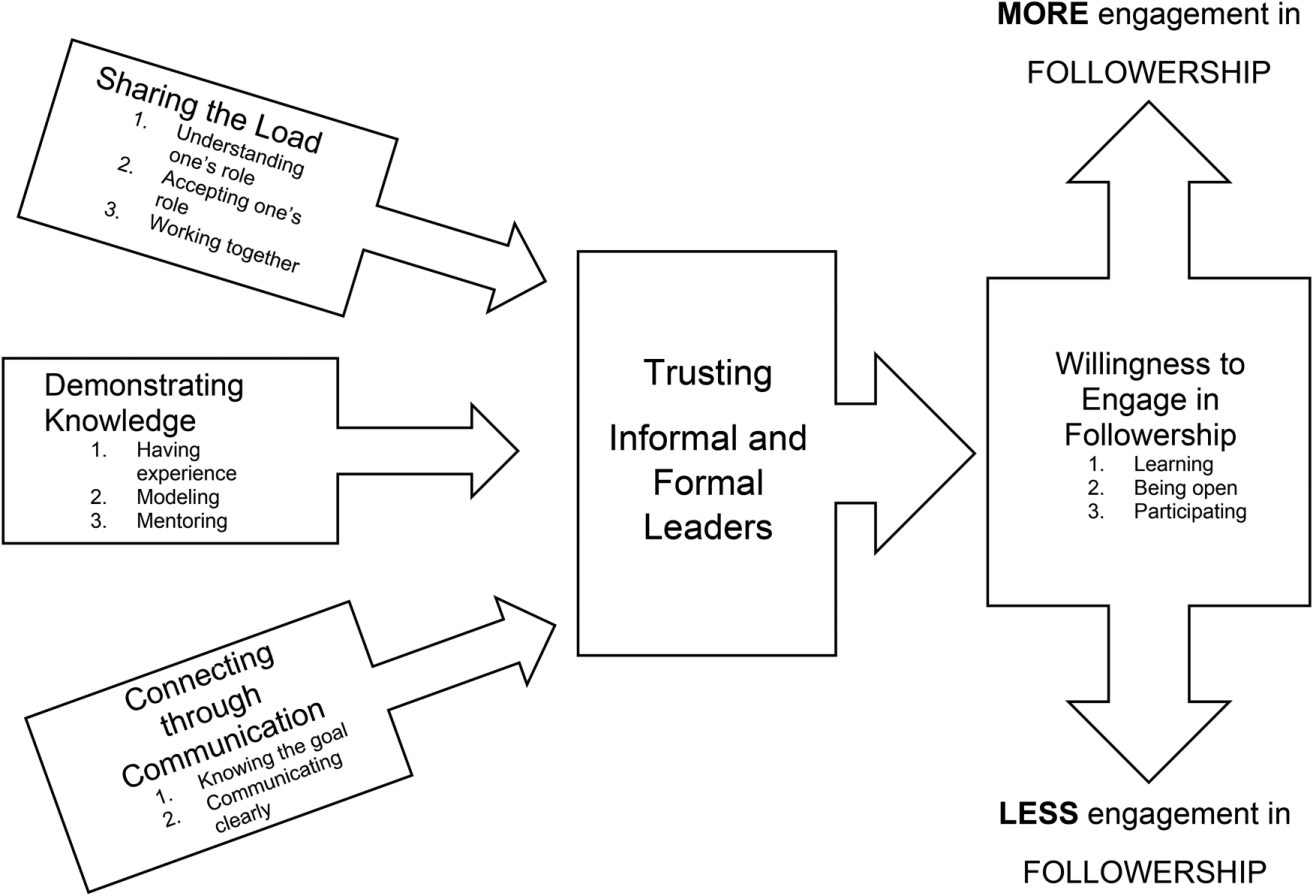
Followership_as_Trust_Concept_Model Revision.docx.

The conceptual model, titled Followership as Trust in Acute Care Nursing Teams, is illustrated in Figure 1. The willingness of team members to engage was grounded in trusting informal and formal leaders. Participants were more likely to engage in following if they trusted the team members to share the load, demonstrate knowledge, and connect through communication. Conversely, if participants believed that team members were not sharing the load, demonstrating knowledge, or connecting through communication, they were less willing to engage in following.

### Sharing the load

Several focused codes merged to support the creation of the category sharing the load and the subcategories understanding one's role, accepting one's role, and working together. Sharing the load was defined as being part of the team, agreeing on a shared goal, and implementing a shared plan. Within the team, everyone was expected to share the load – to work together to care for the team's patients. Participants in this study worked together in teams which creates the context for, and guides, their understanding of followership.

#### Understanding one's role

Although ten of the eleven participants had never heard the term followership prior to participating in the study, all intuitively understood that it was integral to effective team function and involves a leader and the people who get the work done - the followers. Despite not having previous experience with or a language related to followership, participants were able to articulate their understanding of followership, and how they enacted the role. As well, there was overlap and consistency in participants’ perspectives.

I feel it's important that every team member does have their own specific role. So that tasks do get completed in a timely manner…I feel like a follower should also understand their role prior to getting into it. They understand that they are under the umbrella of somebody else. So, somebody that has a little more experience and knowledge and that they are adjustable to…whatever that unit requires from them (P6).

#### Accepting one's role

Participants noted that a large part of sharing the load was dependent on followers accepting what needed to be done, accepting they were not in the leader role, and accepting and following the directions that were given to complete the task.

…I feel like all nurses, [of all levels of] experience need to really understand that everyone was in that same boat … We were all followers whether we recognized it, or we didn’t…I feel like every level of nursing needs to recognize that it's okay to be in that position, to be a follower (P6).

#### Working together

When team members do not work well together, they risk not feeling part of the team and miss a sense of accomplishment: “being a part of that team, and you’re helping…feeling helpful…feeling accomplished…being part of that group dynamic…it definitely makes you feel good…I think working together too helps us be cohesive together…make good choices for our patients…” (P3). A team functioned best when every member did their part to work together: “…everyone works together because it's multiple members of the team to one patient…it's truly based on…how well your team is functioning…” (P10). Team members who worked together demonstrated followership, understanding and accepting their roles to complete the goal set by the team.

### Demonstrating knowledge

Participants described demonstrating knowledge as having knowledge and being able to support that knowing with evidence, policies, or procedures. Participants also noted that demonstrating knowledge was informed by experience, was backed up by evidence, was current, and changed as new evidence was available. Having team members who demonstrated specialized knowledge was integral to safely and effectively completing tasks and working together. Participants also acknowledged that they were reluctant to follow those team members who did not use evidence to guide their practice: “*…*if they give answers that they can’t back up with policy or medical knowledge then sometimes I’m not quite as willing to follow what they say*”* (P4). Three sub-categories having experience, modelling, and mentoring contributed to the creation of the demonstrating knowledge category.

#### Having experience

Having experience was deemed more important than having credentials; it reflected years of practice on the unit and a familiarity with unit and hospital procedures. As these participants explained, they looked to team members who had more experience for guidance: “*…*she's been here the longest, she has the most experience in this department and so…she's taken on that role…no one's ever said to her…you’re the charge nurse or you’re the clinical coordinator…and she definitely doesn’t hold that title formally…*”* (P2) and “…they’re usually more experienced so that I go with them, they’re kind of guiding how my practice is” (P11). Having experience was a definite asset when demonstrating knowledge, with the caveat that the experience be backed up with evidence, policies, or procedures.

#### Modelling

Demonstrating knowledge was exhibited through role modelling, an informal relationship whereby one nurse admired another and emulated their behaviour. This modelling was an important process and guided novice followers in their development as nurses. Participants highlighted the importance of a team member's behaviour: “…they…lead by example…they help out too. And those things…make me want to follow their…example and their lead” (P4). Conversely, participants were not as likely to follow a team member whose behaviours were incompatible with or diverged from team expectations. Having experience and modelling were interwoven and integral to demonstrating knowledge.

#### Mentoring

Participants emphasized the importance of new nurses finding someone to formally mentor them. Demonstrating knowledge in a formal mentorship role was a process that participants understood as important and was a role that a nurse grows into: “…when you’re a follower…you…take those pieces…like what you like about someone that you’re following…and be a mentor yourself…and say, oh I like this piece, I like that piece…maybe I can…implement some of those things” (P3). Finding a mentor was encouraged by participants, especially for new nurses learning to follow: “…find a mentor or a leader or a teacher…then you emulate their practice” (P10). Mentoring facilitated demonstrating knowledge by passing along information, practice, and skills and should be enacted at all levels of a nurse's career, either as mentee or mentor.

### Connecting through communication

Communication was fundamental to followership and was essential to the cohesiveness of the team, increased quality of care, and job satisfaction. Communication was articulated by participants as an exchange of ideas and knowledge in a respectful manner that involved listening to others and understanding what was being communicated. Two sub-categories knowing the goal and communicating clearly supported the category connecting through communication. The team could not complete the task and meet expectations if they did not connect via communication: “…because you don’t want to…assume anything that you’re gonna be doing because that other person might be doing it. And in a…life-threatening situation, any time that you can save is vital to that person's life” (P9). A lack of communication within the team led participants to feel unwilling to follow and decreased the efficacy of the team: “I will never follow someone who is more like a dictator” (P10).

#### Knowing the goal

The nurses in this study believed that followers on nursing teams must endorse the common goal of providing the highest quality of care. An important function of the team lead was to ensure that team goals were clearly communicated: “well it's [the responsibility of the leader] to make sure that everyone is aware of what that goal is and that everyone is…working…towards that goal in the most effective way…so it's really to…focus the group…to what the end goal is going to be” (P2). To follow effectively, the team must understand the goal. Connecting through communication strengthened group cohesion and when the goal was not clear, the team had difficulty working together: “I struggle [to follow] leaders [team members] who just give direction…because maybe they’re given direction at a higher level without questioning the background” (P7) and “…I feel like…if I wasn’t given a…clear…instruction…I will never follow” (P9).

#### Communicating clearly

Communicating clearly was articulated by participants as essential for effective following, understanding one's role, and working together: “…really good communication with those around….and knowing what your role is in the group dynamics so that…your role is done properly” (P11). Having clear directions was an indispensable element of follower-leader communication and effective following. Communicating reiterated the shared goal and strengthened connections between team members. This was especially true when the rationale for the goal or the direction was also clear: “…explaining why [a] change [is necessary] or why we’re being asked to do the specific task is, I think, critical…and basically that there's some benefit to the change, either to the practice or to patient care that we’re providing” (P7). When participants understood their role, they were better prepared and able to follow and function to their fullest capacity on the team.

### Trusting informal and formal leaders

The core category, trusting informal and formal leaders, was developed from constant comparison of initial and focused codes. The four categories sharing the load, demonstrating knowledge, connecting through communication, and willingness to engage emerged as an integral and continuous process in the construction of a preliminary understanding of followership in nursing. Participants noted that their decision to follow was based on their positive assessment of team members including informal and formal leaders’ understanding of the role, their use of evidence-based knowledge, and that informal and formal leaders communicated clearly which facilitated willingness to engage. RNs’ willingness to follow team members including an informal and formal leader was based on trust, which was developed over time and became the thread that connected the categories.

Trust was necessary to effective team function and was reciprocated between and among followers, and leaders. Participant 2 summarized the importance of trust in nursing teams:

…your fellow followers are kind of like the make it or break it thing that makes it easier to follow…the leader…is a big factor in what it's like to follow someone but also your fellow followers…are a big factor too…it's easier to…work towards that goal if your fellow followers are…of the same like mind, work the same…take care of each other, that kind of stuff. Because, working on the floor…it's your team of your people who make it or break it for you…whether you’re in a leadership role or not, the people who are around you, you know that…it's going to be a good shift or not a good shift…you know if you can handle whatever comes through the door if your fellow co-workers…are a good team.

Sharing the load was essential among followers and for each team member to work to their highest potential. In the absence of sharing of the load, participants were less motivated to engage in following. Participants were more apt to follow a leader who demonstrated knowledge and could support that knowledge with evidence. Connecting through communication was integral to the willingness to engage especially regarding making sure team members had the knowledge they needed to complete the task and meet the goals of the team.

### Willingness to engage

Willingness was described as feeling ready or inclined to engage in following activities: “If I’m following others and they’re confident and honest and transparent and trustworthy and just generally a nice person, I want to follow them more” (P8).

I like to approach work with a really positive and optimistic mindset, and I understand that change is difficult and with COVID…there has been a lot of change and some staff members are really…not opposing the change, but just have a negative mindset about the change. And I think that can be really deterring to both followership and teamwork…because that can…lead to…you’re just not miserable, but…you just don’t approach work with the same passion that you should…which deters the teamwork…then you’re not likely to follow the mission set out by the leader (P1).

Also, participants commented that they were less willing to engage in followership if they did not feel respected within the team or the organization. The willingness to follow arose from the need to work together as a team and was maintained by continuing involvement in the processes of learning, being open, and participating.

#### Learning

Participants noted that learning was about being willing to change and be open to new experiences and was integral to followership: “I hope they would be open to new learning and…like to learn from the person that they’re following…I would hope that they wouldn’t be…closed off or opinionated” (P5). Learning takes time and when support was provided by leaders and the organization (e.g., funding and time off to attend continuing education events), participants were more willing to take the time to attend courses.

I guess when I think of the…example, like something within the organization that made it easier for myself and every other nurse to follow as far as…practicing and learning and feeling okay…was that there was adequate time provided, adequate pay provided to do the preparation work…so that if you learned one way versus another you…could access the different learning styles. (P8)

Lifelong learning was essential to positive patient care outcomes and directly affects nursing teams’ primary goal. Taking part in learning events enabled RNs to function at their highest potential and in turn increased following.

#### Being open

Participants did not consider being open to new ideas or practices as being an empty vessel, but rather as being open to new experiences and the potential to improve. When asked to describe how they felt about setting aside their beliefs to consider a new idea or a proposed change, Participant 1 explained that their feelings changed from negative to positive:

…initially for me…it was a negative feeling because I wasn’t used to it. However, after the end, when I started debriefing with…my instructor and my preceptor, I realized that it was a really positive experience, a setting aside my beliefs to provide…the holistic sense of care that [was] required for my patient and being able to…step up outside of my shoes into their shoes made me…feel…I don’t know…like it was rewarding…to be able to do that. (P1)

Followers affect their team members’ attitudes toward openness. As one participant commented, “being openminded and receptive to what's being asked and ideally trying to motivate others to align with what's being asked” (P7). Being open to caring for others in a manner that supports their needs, being open to learning in new situations and contexts, and being open to listening to a new idea increased their willingness to follow and their team cohesion.

#### Participating

Participating was an action which was demonstrated by helping, observing, following directions, and working together; it relied on synergy, cohesion, and teamwork. “I see the followers as…the people carrying out the actions, the doers” (P8). “They are the ones who work to achieve the leader's vision or goal” (P1). A willingness to engage was integral to following directions, however when participants did not like a change or were not supportive of the leader or team members they were not motivated to participate.

I feel like what I’ve seen sometimes in practice is…co-workers that may…team up together to think that we don’t like this change, we don’t want it to succeed. We’re doing…everything we can to…shine negative light on it, or to not help others…follow…by kind of nit picking or…just not doing what's meant to be done, what was asked of them and kind of going against the leader. (P8)

Participating worked hand in hand with being open and learning and was inherent to a willingness to engage.

## Discussion

The concept of followership first appeared in the nursing literature in the middle of the last century. In the intervening years, the topic resurfaced periodically in editorials, opinion pieces, and three research studies whose authors endorsed its importance in nursing practice and decried the lack of scholarship regarding followership in the nursing context (Honan et al., 2022). Findings from this study contribute new disciplinary knowledge about followership. RNs were more likely to follow team members who they trusted to share the load, demonstrate knowledge, and connect through communication. Conversely, when these things were absent trust decreased as did their willingness to follow.

Like followership, the concept of trust in nursing is not new (Brunetto et al., 2013; Johns, 1996; Meize-Grochowski, 1984). Participants in this study underscored the importance of trust to leadership, followership, and team function. The degree to which RNs trusted informal and formal leaders directly affected how they felt about coming to work, patient care, and whether they were willing to engage in following. Soderberg and Romney (2022, p. 173) emphasized the importance of trust “across organizations, industries, and cultures … [as] a leader's primary means of influence”.

Although most participants were unfamiliar with the concept of followership before the interview, they intuitively seemed to understand what Kelley (1992) pointed out thirty years ago – namely, that followers and leaders were parts of the same phenomenon and the two cannot exist independently from the other. Participants’ construction of followership and innate understanding of the follower-leader dynamic was undoubtedly shaped by their understanding and experience of team nursing. Nursing teams work collaboratively toward a common goal, share responsibility, support each other, and provide peer mentorship. Team cohesion and support was based on mutual trust (Kalisch et al., 2009). Teams that function well together understand their roles (Kaiser & Westers, 2018) and engage in “interdependent collaboration, open communication, shared decision-making, and generate value-added patient, organizational and staff outcomes” (Xyrichis & Ream, 2008, p. 232). In other words, effective followership.

In their study of followership, Carsten et al. (2010) concluded that followership was socially constructed and was influenced by an individual's schema (beliefs, perceptions, experiences), behaviours, and the context of leadership and organization climate. RNs in the current study spoke of being in the follower role as a new graduate and how they solidified their beliefs (schema) about what comprises safe, competent, and evidence-based nursing practice. Participants’ beliefs that sharing the load, demonstrating knowledge, and connecting through communication played an integral part in whether they were willing to engage in followership. These findings have important implications for followership regarding the relationship between followers and leaders and how followership affected team function. Equally important to participants were expressions of respect from the organization. Findings from this study indicated that trust and respect within the organizational culture were imperative to the willingness to engage in followership and to teamwork.

### Recommendations

Research on followership in nursing has the potential to advance follower's and leader's understanding and performance of their roles (Uhl-Bien et al., 2014). The limited number of studies of followership in nursing highlight a gap in our knowledge about the leader-follower dynamic and underscores the need for more research on the topic. The findings of this study suggest the need for a concept analysis of followership to define its boundaries and identify its relationship to trust and team function. A clear, conceptual definition of followership could inform development opportunities for formal leaders in an organization as well as orientation modules for new nursing graduates and nurses new to a unit or hospital. From an organizational perspective, consistency in messaging regarding the role of nurses, whether as followers or leaders, is essential to shaping organizational policies and practices; communication; and patient care outcomes (American Nurses Credentialing Center, n.d.).

The findings from this study are potentially of interest to nurse educators as they update their curricula. Undergraduate nursing education has focused almost exclusively on leadership, with some content directed to the topic of followership, despite followership and leadership being interdependent and essential to teamwork. Nurses are followers and leaders throughout their careers and preparing them to function effectively in both roles ultimately affects patient care. Integrating the concept of followership would provide nursing students with a language and the foundational knowledge needed as they embark on their careers. A practice recommendation would be that education sessions for practicing nurses include the topic of followership especially in relationship to leadership and interprofessional collaborative practice. Nurses participate in following and leading roles throughout their careers and a fulsome understanding of followership in nursing through academic institutions and workplace education benefits nurses, organizations, and healthcare systems (DiRienzo, 1994; Lopez & Freeman, 2018; Whitlock, 2013).

### Limitations

Limitations of this study include: (1) participants’ lack of familiarity with the concept of followership, which may have affected their ability to reflect upon and describe their understanding of the topic; (2) data collection occurred at a single acute care site; (3) the ongoing effects of the COVID pandemic and related stressors on RNs’ understanding and experience of followership; and (4) the lack of diversity in participants’ sex and gender.

## Conclusion

This study highlights the need for a holistic understanding of followership in nursing and its contribution to the leader-follower dynamic. Followers and leaders are interdependent, their relationship is synergistic, they are not mutually exclusive, and both roles are equally important. In the current study, the findings highlight the need for more research on followership in nursing and its relationship to trust, teamwork, and organizational culture. The role of followers must be acknowledged as legitimate and as essential to the follower/leader dyad. Without followers, teams, patient care units, and healthcare organization cannot function to their fullest capacity. Education on followership in nursing, that begins in nursing programs and is encouraged post-licensure, is needed to support effective followership for all nurses.
